# Two dopamine receptors play different roles in phase change of the migratory locust

**DOI:** 10.3389/fnbeh.2015.00080

**Published:** 2015-03-31

**Authors:** Xiaojiao Guo, Zongyuan Ma, Le Kang

**Affiliations:** ^1^Beijing Institutes of Life Sciences, Chinese Academy of SciencesBeijing, China; ^2^State Key Laboratory of Integrated Management of Pest Insects and Rodents, Institute of Zoology, Chinese Academy of SciencesBeijing, China

**Keywords:** *Locusta migratoria*, neurotransmitter, gregarious, solitary, RNA interference, phenotypic plasticity, behavior, phase change

## Abstract

The migratory locust, *Locusta migratoria*, shows remarkable phenotypic plasticity at behavioral, physiological, and morphological levels in response to fluctuation in population density. Our previous studies demonstrated that dopamine (DA) and the genes in the dopamine metabolic pathway mediate phase change in *Locusta*. However, the functions of different dopamine receptors in modulating locust phase change have not been fully explored. In the present study, DA concentration in the brain increased during crowding and decreased during isolation. The expression level of dopamine receptor 1 (Dop1) increased from 1 to 4 h of crowding, but remained unchanged during isolation. Injection of Dop1 agonist SKF38393 into the brains of solitary locusts promoted gregarization, induced conspecific attraction-response and increased locomotion. RNAi knockdown of Dop1 and injection of antagonist SCH23390 in gregarious locusts induced solitary behavior, promoted the shift to repulsion-response and reduced locomotion. By contrast, the expression level of dopamine receptor 2 (Dop2) gradually increased during isolation, but remained stable during crowding. During the isolation of gregarious locusts, injection of Dop2 antagonist S(–)-sulpiride or RNAi knockdown of Dop2 inhibited solitarization, maintained conspecific attraction-response and increased locomotion; by comparison, the isolated controls displayed conspecific repulsion-response and weaker motility. Activation of Dop2 in solitary locusts through injection of agonist, R(-)-TNPA, did not affect their behavioral state. Thus, DA-Dop1 signaling in the brain of *Locusta* induced the gregariousness, whereas DA-Dop2 signaling mediated the solitariness. Our study demonstrated that Dop1 and Dop2 modulated locust phase change in two different directions. Further investigation of *Locusta* Dop1 and Dop2 functions in modulating phase change will improve our understanding of the molecular mechanism underlying phenotypic plasticity in locusts.

## Introduction

Phenotypic plasticity contributes to the adaptation of animals, in vertebrates and invertebrates, to environments at behavioral, physiological, and morphological levels (West-Eberhard, 2003; Moczek, 2010). Behavioral phenotypes of animals are affected and modified by external and internal factors (Caspi and Moffitt, 2006). Internal factors allow animals to acclimate during environmental changes to trigger the evolution of biological traits (West-Eberhard, 2003; Grether, 2005). For instance, the migratory locust, *Locusta migratoria*, one of the important economic pest species, shows density-dependent polyphenism, gregarious phase and solitary phase (Uvarov, 1966). Locusts in gregarious phase are active for aggregation and swarming, whereas locusts in solitary phase tend to avoid contact with conspecifics and exhibit weak motility (Uvarov, 1966; Pener and Simpson, 2009). Molecular and genetic mechanisms underlying behavioral changes between solitary and gregarious phases of *Locusta* have been revealed (Kang et al., 2004; Guo et al., 2011; Ma et al., 2011; Wu et al., 2012). Among these vital findings, we confirmed that key genes in the catecholamine metabolic pathway are active in gregarious locusts; we also found that DA concentration in the brain of gregarious locusts is higher than that of solitary locusts. Moreover, the activation of dopamine pathway through injection of DA and its receptor agonists promotes behavioral phase change from solitariness to gregariousness. By contrast, specific RNAi knockdown of *pale*, *henna*, *vat 1*, dopamine receptor 1 (*Dop1*) in dopamine pathway in gregarious locusts results in their behavioral changes toward solitary phase (Ma et al., 2011). A recent study has found that miR-133 also modulates behavioral phase change by inhibiting DA synthesis in aggregation of *Locusta* (Yang et al., 2014). Transcriptome analysis has further revealed that neurochemicals including DA may be involved in behavioral phase changes through G protein-coupled receptor (GPCR) pathways (Chen et al., 2010).

The desert locust, *Schistocerca gregaria*, another locust species, also exhibits reversible changes between solitary and gregarious phases (Pener and Simpson, 2009). In contrast to *Locusta* showing quick solitarization and slow gregarization (Guo et al., 2011; Wang and Kang, 2014), *Schistocerca* exhibits quick gregarization and slow solitarization (Roessingh and Simpson, 1994). Previous studies confirmed the alternative mechanism underlying phase change between these two locust species. In particular, *Schistocerca* shows behavioral change from solitary to gregarious phase when serotonin (5-HT) is applied to the thoracic ganglia of this locust (Anstey et al., 2009; Rogers et al., 2014). Likewise, cAMP-dependent protein kinases (PKA) is proposed to link with 5-HT signaling and mediates gregarization in *Schistocerca* (Ott et al., 2012). However, attraction/avoidance behavior, which is one of the most important behavioral traits in aggregation, is not affected by injection of 5-HT in the hemocoel of *Schistocerca* from another laboratory (Tanaka and Nishide, 2012). Conversely, injection of 5-HT and its receptor agonist in the brain of *Locusta* leads to the behavioral change to solitary phase and inhibits gregarization in crowding (Guo et al., 2013). In another study, the solitarization of *Schistocerca* is modulated by DA, in which DA amount in the thoracic ganglia increases during isolation of the gregarious locusts; solitary-like behavior is also induced by DA injection into the hemocoel of gregarious *Schistocerca* (Alessi et al., 2014). By contrast, gregarious-like behavior is triggered when non-selective antagonist of dopamine receptors, fluphenazine is injected into the thoracic hemocoel of gregarious locusts isolated for 1 h (Alessi et al., 2014). Basing on the results, Alessi et al. (2014) concluded that the solitary behavior of *Schistocerca* is promoted by DA.

In *Locusta* and *Schistocerca*, the contrasting roles of DA are elucidated through systematic pharmacology (Ma et al., 2011; Alessi et al., 2014). In *Locusta*, the roles of DA and its related genes have been confirmed through systematic pharmacological intervention and RNAi knockdown. In *Schistocerca*, the function of DA has been verified by systematic injection of chemicals into the hemocoel. In the central nervous system, the brain integrates information from the sensory system in the head and transmits sensory information to the thoracic ganglia for motility; the thoracic ganglia mainly regulate general motor programs (Schaefer and Ritzmann, 2001; Wessnitzer and Webb, 2006; Zill, 2010). Thus, systematic injection of neurochemicals into the hemocoel may interfere with specific roles in the brain and the thoracic ganglia. However, the specific roles of DA and corresponding receptor subtypes in the brain of *Locusta* and *Schistocerca* during phase change remain poorly understood.

DA modulates diverse behaviors in vertebrates and invertebrates through varied receptors in specific regions of the central nervous system (Romanelli et al., 2010; Mustard et al., 2012). In vertebrates, five dopamine receptor subtypes have been identified; these subtypes have been further classified into two major groups, namely, D1-like (D1 and D5) and D2-like (D2, D3, and D4) receptors (Romanelli et al., 2010). In invertebrates, specifically in insects, four subtypes of dopamine receptors, which generally named as D1-like dopamine receptor (Dop1), invertebrate type dopamine receptors (INDRs or Dop2), D2-like dopamine receptor (Dop3), and DopEcR, have been identified (Mustard et al., 2005; Srivastava et al., 2005; Watanabe et al., 2013). Among these subtypes, D1-like dopamine receptor, INDRs, and DopEcR up-regulate intracellular cAMP levels, whereas D2-like dopamine receptor decreases intracellular cAMP levels (Beggs et al., 2005; Mustard et al., 2005; Srivastava et al., 2005; Verlinden et al., 2015). Indeed, these dopamine receptor subtypes show significant differences in sequence, structure, and function in animals (Mustard et al., 2005; Romanelli et al., 2010); as such, the opposite effects of DA on the regulation of phase change of the two locust species through different receptor subtypes may be a very interesting phenomenon.

In this study, the levels of DA and the expression of the two dopamine receptors in the brains were determined to elucidate their roles in phase change of *Locusta*. Our findings clarified the roles of DA and its receptors in gregarization and solitarization of *Locusta*. Specific agonist and antagonist combined RNAi knockdown were also used to distinguish the roles of *Locusta* Dop1 and Dop2 implicated in behavioral phase change of *Locusta*. We confirmed that DA-Dop1 signaling in the brain modulated the gregarization of *Locusta*, whereas DA-Dop2 signaling mediated the solitarization of *Locusta*. Furthermore, DA-Dop1 mediated conspecific attraction behavior and increased motility. By contrast, DA-Dop2 signaling induced avoidance behavior and reduced motility during phase change.

## Materials and methods

### Animal husbandry

Experiments were performed using fourth-stadium migratory locust (*L. migratoria*) from the solitary and gregarious colonies maintained in the Institute of Zoology, Chinese Academy of Sciences, Beijing, China. Gregarious locusts were cultured in large boxes (40 × 40 × 40 cm^3^) at a density of 500–1000 locusts per container. Solitary locusts were obtained from the gregarious colony and cultured individually in separate white metal boxes (10 × 10 × 25 cm^3^). These boxes were supplied with charcoal-filtered compressed air. Gregarious and solitary locusts were maintained for at least three generations before the experiments were conducted. Gregarious and solitary colonies were maintained in a 14 h light/10 h dark cycle at 30 ± 2°C and fed with fresh wheat seedlings and bran (Kang et al., 2004).

### Measurement of DA levels in the brain of the migratory locust

DA in the brain (without optic lobe) of *Locusta* was quantified through reverse-phase high-performance liquid chromatography (HPLC) and electrochemical detection (ECD), as previously described (Guo et al., 2013). DA levels were quantified by referring to external standards. A standard curve was generated through serial dilutions of standard solution containing DA (Sigma-Aldrich, USA).

### Phylogenetic analysis of dopamine receptors

To confirm the receptor subtypes, we cloned sequences of dopamine receptors by referring to putative sequences in genome and transcriptome database of *Locusta* (Wang et al., 2014). We uploaded the nucleotide sequences of *Locusta* Dop1 and *Locusta* Dop2, and acquired the corresponding Genbank accession numbers (*Locusta* Dop1, KP780182; *Locusta* Dop2, KP780183). The other sequences for phylogenetic analysis were downloaded from NCBI databases. Multiple sequence alignments of 300 amino acids (including TM2 to TM7) in these insect dopamine receptors were performed in Clustal W and curated in MEGA 5.34 (Tamura et al., 2011) to classify the subtypes of dopamine receptors. Neighbor-joining analysis was performed using MEGA 5.34 with 1000 bootstrap replicates. The following Genbank accession numbers (NCBI) were used in this study: *Acromyrmex* Dop1, EGI58704; *Acromyrmex* Dop2, EGI63314; *Acyrthosiphon* Dop1, XP_001947683; *Acyrthosiphon* Dop2, XP_003241369; *Aedes* Dop1, AFB73766; *Apis* Dop1, NP_001011595; *Apis* Dop2, NP_001011567; *Apis.f* Dop1, XP_003697539; *Apis.f* Dop2, XP_003696107; *Bombus.i* Dop1, XP_003487004; *Bombus.i* Dop2, XP_003490584; *Bombus.t* Dop1, XP_003401071; *Bombus.t* Dop2, XP_0034019c; *Bombyx* Dop1, NP_001108459; *Bombyx* Dop2, NP_001108338; *Camponotus* Dop1, EFN72198; *Camponotus* Dop2, EFN69323; *Drosophila* Dop1, P41596; *Drosophila* Dop2, Q24563; *Gryllus* Dop1, BAM15634; *Gryllus* Dop2, BAM15635; *Harpegnathos* Dop1, EFN84948; *Harpegnathos* Dop2, EFN84440; *Manduca* Dop1, AEU17117; *Megachile* Dop1, XP_003708644; *Megachile* Dop2, XP_003704778; *Nasonia* Dop1, XP_001606438; *Nasonia* Dop2, NP_001155849; *Tribolium* Dop1, XP_971542; *Tribolium* Dop2, XP_972779; *Apis* Dop3, NP_001014983; *Drosophila* DDR2, NP_001014760; *Nasonia* Dop3, XP_001602510; *Pediculus* Dop3, XP_002426923; *Tribolium* Dop3, EFA02832; *Agrotis* DopEcR, AGN74919; *Drosophila* DopEcR, AAF47893; and *Gryllus* DopEcR, BAM15638.

### Isolation and crowding treatments of the migratory locust

Fourth-stadium gregarious locusts were isolated and individually reared as solitary locusts. After 1, 4, 16, or 32 h of isolation, the brains of isolated locusts were dissected and immediately placed in RNAlater solution (Ambion, Austin, Texas, USA) for quantitative real-time PCR (qRT-PCR) analysis. The brains of gregarious locusts were sampled as the control group for isolation treatment. Meanwhile, 10 solitary locusts at the fourth-stadium were introduced to an optic perplex-made box (10 × 10 × 10 cm^3^) and allowed to live with 20 gregarious locusts at the same stadium. After these solitary locusts were allowed to stay with the gregarious locusts for 1, 4, 16, or 32 h, the brains of the crowded locusts were dissected and immediately placed in RNAlater solution for subsequent qRT-PCR analysis. The brains of solitary locusts were sampled as the control group for crowding treatment. All of the locusts were sampled at the same time point for eight biological replicates, and equal numbers of male and female locusts were sampled per replicate.

### RNA preparation and qRT-PCR assay

Total RNA was extracted from brain tissues by using RNAeasy mini kit in accordance with the manufacturer's protocol (QIAGEN, Hilden, Germany). DNase was applied to eliminate DNA contamination in RNA samples. To analyze the expression levels of the target genes, we reversely transcribed 2 μg of total RNA in each sample by using MMLV reverse transcriptase (Promega, Madison, USA, Madison, USA) in accordance with the manufacturer's instructions. PCR amplification was conducted in Roche Light Cycler 480 using RealMaster-Mix (SYBR Green) kit (Tiangen, Beijing, China). Amplification was initiated by incubation at 95°C for 5 min, followed by 40 cycles at 95°C for 20 s, 58°C for 20 s, and 68°C for 20 s. Melting curve was detected to confirm the amplification specificity of target genes. Table 1 lists the primers used in qRT-PCR assay. We also screened the expression levels of housekeeping genes and then analyzed the expression levels of the target genes. The most stably expressed gene *RP*–*49* was chosen as reference to normalize and calculate the expression levels of the target genes (Ma et al., 2015).

**Table 1:** **Primer sequences for quantitative RT-PCR**.

**Genes**	**Forward primer**	**Reverse primer**
*Dop1*	GCGCATCGGCAACCTCTTC	GATCCAGGTGTCGCAGAAC
*Dop2*	GTTACAATAATTTCCGTTCC	GGCTTTACACCGTTCTCAT
*RP*–*49*	CGCTACAAGAAGCTTAAGAGGTCAT	CCTACGGCGCACTCTGTTG

### Behavioral pharmacology

#### Behavioral pharmacology of solitary locusts

We injected SKF38393, a specific agonist of D1-like dopamine receptor (Feng et al., 1996; Titlow et al., 2013), in the brains of solitary locusts to determine the role of *Locusta* Dop1 in modulating behavioral transition from solitary to gregarious phase. All injection procedures were performed under a stereo-dissection microscope using a NANOLITER injector 2000 (World Precision Instruments, Sarasota, FL, USA) with a glass micropipette tip (Guo et al., 2013). Brain tissue dissection was performed as described in previously published methods (Guo et al., 2013). After SKF38393 (5 mM × 69 nL; Sigma-Aldrich, USA) was injected, each of the solitary locusts was placed in separate solitary-rearing cages to live individually for 4 h or 10 solitary locusts were allowed to live with 20 gregarious locusts in a plastic box (10 × 10 × 10 cm^3^) for 4 h before behavioral assay. The control group received the same volume of saline before behavioral assay was conducted.

We then injected R(-)-2,10,11-trihydroxy-*N*-propyl-noraporphine hydrobromide hydrate [R(-)-TNPA] (5 mM × 69 nL; Sigma-Aldrich, USA), a D2-like receptor selective agonist (Keating and Orchard, 2004; Marg et al., 2004), in the brains of solitary locusts to determine the role of Dop2 in modulating behavioral transition. After the agonist was injected, each of the fourth-stadium solitary locusts was placed in separate cages for 60 min to live individually before behavioral assay was conducted.

#### Behavioral pharmacology of gregarious locusts

We injected SCH23390 (5 mM × 69 nL; Sigma-Aldrich, USA), a specific antagonist of D1-like receptor (Kokay and Mercer, 1996; Titlow et al., 2013) in the brains of gregarious locusts before behavioral assay was performed to determine the role of Dop1 in gregarization of solitary locusts. After the antagonist was injected, fourth-stadium gregarious locusts were reared with other gregarious locusts for 1 h before behavioral assay was conducted.

To evaluate the role of Dop2 in the solitarization of gregarious locusts, S(-)-sulpiride (5 mM × 69 nL; Sigma-Aldrich, USA), a specific antagonist of Dop2 (Feng et al., 1996), was injected in the brains of gregarious locusts before behavioral assay was performed. After the antagonist was injected, fourth-stadium gregarious locusts were placed in separate solitary-rearing cages to live individually for 15, 30, or 60 min before behavioral assay was conducted. All of the control groups received the same volume of saline before behavioral assay was carried out.

### RNA interference of dopamine receptors

Double-stranded RNA (dsRNA) of green fluorescent protein (GFP), *Dop1* and *Dop2* were prepared using the T7 RiboMAX Express RNAi system (Promega, Madison, USA) according to the manufacturer's instructions. We then selected the fragment without homologies with other genes in the genome database to avoid non-specificity in RNAi knockdown. Table 2 lists the primers for dsRNA preparation. We directly injected 36 ng of dsRNA into the brains of the fourth-stadium gregarious locusts. After injection for 72 h, the ds*DA1*-injected gregarious locusts were directly assayed. The ds*DA2*-injected gregarious locusts were directly assayed or lived individually in solitary-rearing cages for 15, 30, or 60 min before behavioral assay was conducted. The effects of RNAi on the relative mRNA level were detected through qRT-PCR after dsRNA was injected for 72 h.

**Table 2:** **Primer sequences for RNAi**.

**Genes**	**Forward primer**	**Reverse primer**
*Dop1*	TCAACGACCTGCTGGGCTA	AAGGGCACCCAGCAGATGA
*Dop2*	TTCGTGCGGATACTGTGCG	AGGCGGACAGTTGGAGACC
*GFP*	CACAAGTTCAGCGTGTCCG	GTTCACCTTGATGCCGTTC

### Behavioral assay

EthoVision system (Noldus Inc., Wageningen, The Netherlands) was used for video recording and data extraction. The arena behavior assay was performed in a rectangular arena (40 × 30 × 10 cm^3^). The wall of the arena is opaque plastic, and the top is clear. One of the separated chambers (7.5 × 30 × 10 cm^3^) contained 20 fourth-stadium gregarious locusts as the stimulus group, and the other end of the chamber with the same dimensions was left empty. Both ends of the chamber were equally illuminated to prevent formation of locust shadows. The floor of the open arena was covered with filter paper to avoid contamination during the behavior assay. The locusts were gently transferred through a tunnel to the arena. Each locust was recorded for 6 min and examined only once (Roessingh et al., 1993; Ma et al., 2011; Guo et al., 2013).

We constructed a binary logistic regression model in SPSS 15.0 to measure and evaluate the phase state of the fourth-stadium solitary and gregarious locusts and to measure and quantify their behavioral phenotype. Eleven different behavioral parameters were expressed as a mixture of behavioral or categorical markers. These markers were acquired as follows: entry frequency in the stimulus area (EFISA, stimulus area was defined as 25% of the arena closest to the stimulus group), latency of first occurrence in the stimulus area (LFOISA), total duration in the area close to the arena wall (TDCW), entry frequency in the area close to the arena wall (EFCW), entry frequency in the region opposite the stimulus area (EFIOSA, the opposite of the stimulus area was defined as 25% of the arena at the opposite end of the stimulus group), latency of first occurrence opposite the stimulus area (LFOIOSA), mean distance to the stimulus group (MDTSG), total distance moved (TDM), total duration of movement (TDMV), frequency of movement (FOM), and attraction index (AI, AI is the extent of tested animals attracted by the stimulus group; AI = total duration in stimulus area-total duration in opposite area). The behavioral parameters of this model were adjusted until the regression model discriminated the two phases at the optimum level according to the following equation: *P*−*sol* = *e*η/(1 + *e*η), where η = β0 + β1·*X*1 + β2·*X*2 + … + βk·*X*k, *X*1, *X*2, …, where *X*k is the behavioral covariates. *P*-*sol* is the probability that the locusts should be regarded as a member of the solitary phase population. The probability value ranges from 1 to 0, where 1 indicates that individuals display solitary behavior and 0 indicates that individuals display gregarious behavior. The most robust indicators (TDM, FOM, and AI) of the phase state were retained in the model. *P*-*sol* was calculated according to the following equation: *P*-*sol* = 2.361 − 0.016 × TDM − 0.172 × FOM − 0.005 × AI (Guo et al., 2013; Ma et al., 2015). This model correctly classified 89.2 and 91.2% of the solitary and gregarious populations, respectively. The model also shares similar features with previous regression models used for binary discrimination of solitary and gregarious locusts (Roessingh et al., 1993; Anstey et al., 2009; Guo et al., 2011; Ma et al., 2011, 2015).

### Statistical analysis

DA content and expression levels of dopamine receptors over a time course of crowding and isolation were analyzed by One-Way ANOVA, followed by *post-hoc* Tukey test for multiple comparisons. The data for behavioral phase change were analyzed by Mann–Whitney *U* (MWU) test because of their non-normal distribution characteristics. Student's *t*-test was conducted to analyze the changes of specific parameters, including TDM and FOM, between saline- and agonist-injected, saline- and antagonist-injected, dsGFP- and ds*Dop1*-injected groups. The changes of TDM and FOM during isolation were analyzed by One-Way ANOVA, followed by *post-hoc* Tukey test for multiple comparisons. The change of parameter AI was analyzed by MWU test. The data were expressed as mean ± standard error of the mean. *P* < 0.05 was considered statistically significant. The probabilistic metric of solitariness (*P*-*sol*) is presented as median values. All statistical data were analyzed with SPSS 15.0 (SPSS Inc., Chicago, IL, USA).

## Results

### Fluctuations of DA level in the locust brains

**Figure 1:**
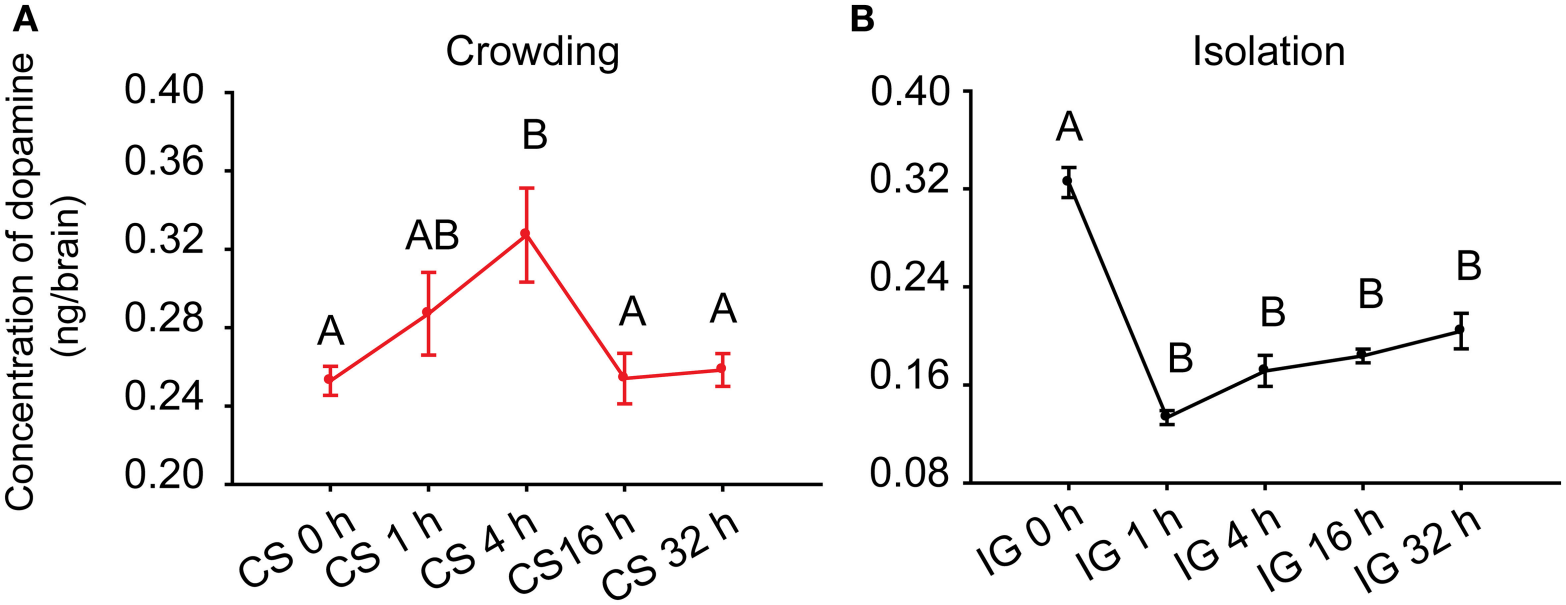
**Dopamine levels in the brains of the migratory locust during the time course of crowding and isolation. (A)** Concentration of DA in the brains of solitary locusts in the time course of crowding. **(B)** Concentration of DA in the brains of gregarious locusts in the time course of isolation. Dopamine levels in crowding and isolation were analyzed by One-Way ANOVA, followed by *post-hoc* Tukey test for multiple comparisons (*n* = 8 for each point, *P* < 0.05). Abbreviations: DA, dopamine; CS, crowding of solitary locusts; IG, isolation of gregarious locusts.

Ma et al. (2011) reported that DA concentration in the brains of gregarious locusts is higher than that of solitary locusts. To determine the association of DA with phase change, we detected its concentration in the brains of *Locusta* during crowding and isolation. During the crowding of solitary locusts (CS), the DA level in the brains increased after 4 h of crowding and decreased thereafter (One-Way ANOVA, *F* = 4.760, *P* = 0.004) (Figure 1A). During the isolation of gregarious locusts (IG), the DA level significantly decreased after 1 h of isolation (One-Way ANOVA, *F* = 40.890, *P* < 0.001) (Figure 1B). These results suggest that the changes of DA levels are possibly related to the behavioral phase change of *Locusta*.

### Expression of dopamine receptors in the locust brains

**Figure 2:**
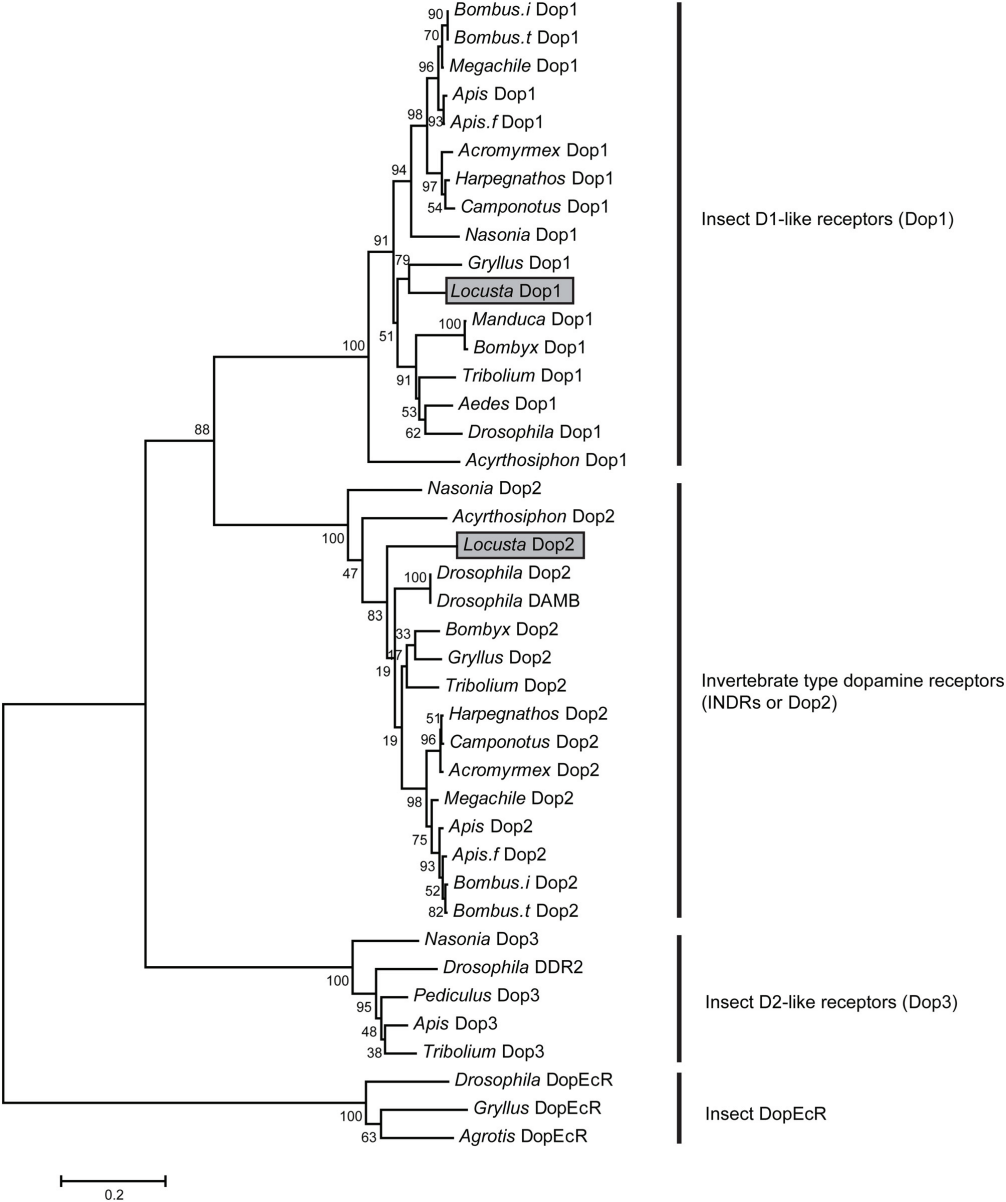
**Phylogenetic analysis of dopamine receptors**. Phylogenetic and molecular evolutionary analyses were conducted using MEGA 5. The dopamine receptors of the migratory locust (*Locusta* Dop1 and Dop2) are indicated by gray bars. Abbreviations: *Acromyrmex, Acromyrmex echinatior; Aedes, Aedes aegypti; Agrotis, Agrotis ipsilon; Apis, Apis mellifera; Apis.f, Apis florea; Bombus.i, Bombus impatiens; Bombus.t, Bombus terrestris; Bombyx, Bombyx mori; Camponotus, Camponotus floridanus; Drosophila, Drosophila melanogaster; Harpegnathos, Harpegnathos saltator; Gryllus, Gryllus bimaculatus; Locusta, Locusta migratoria; Manduca, Manduca sexta; Megachile, Megachile rotundata; Nasonia, Nasonia vitripennis; Pediculus, Pediculus humanus corporis; Tribolium, Tribolium castaneum*.

We cloned two dopamine receptors referring to genome and transcriptome databases (Ma et al., 2011; Wang et al., 2014) to further investigate the role of DA in modulating the behavioral phase change of *Locusta*. A phylogenetic analysis was performed using MEGA5.34 (Tamura et al., 2011) to validate and classify the dopamine receptor subtypes. The results showed that the two orthologous receptors belonged to the two insect dopamine receptor families, namely, D1-like receptors (Dop1) and invertebrate type dopamine receptors (INDRs or Dop2) (Figure 2). The conserved transmembrane (TM) segments of dopamine receptors were analyzed by TMHMM Server 2.0 (Sonnhammer et al., 1998). The full length of *Locusta* Dop1 cDNA encoded seven TM segments, which corresponded to all GPCR TM segments (TM1–TM7 in Supplementary Figure 1). The partial sequence of *Locusta* Dop2 encoded the second to seventh TM segments among all Dop2 TM segments (TM2 and TM7 in Supplementary Figure 2).

**Figure 3:**
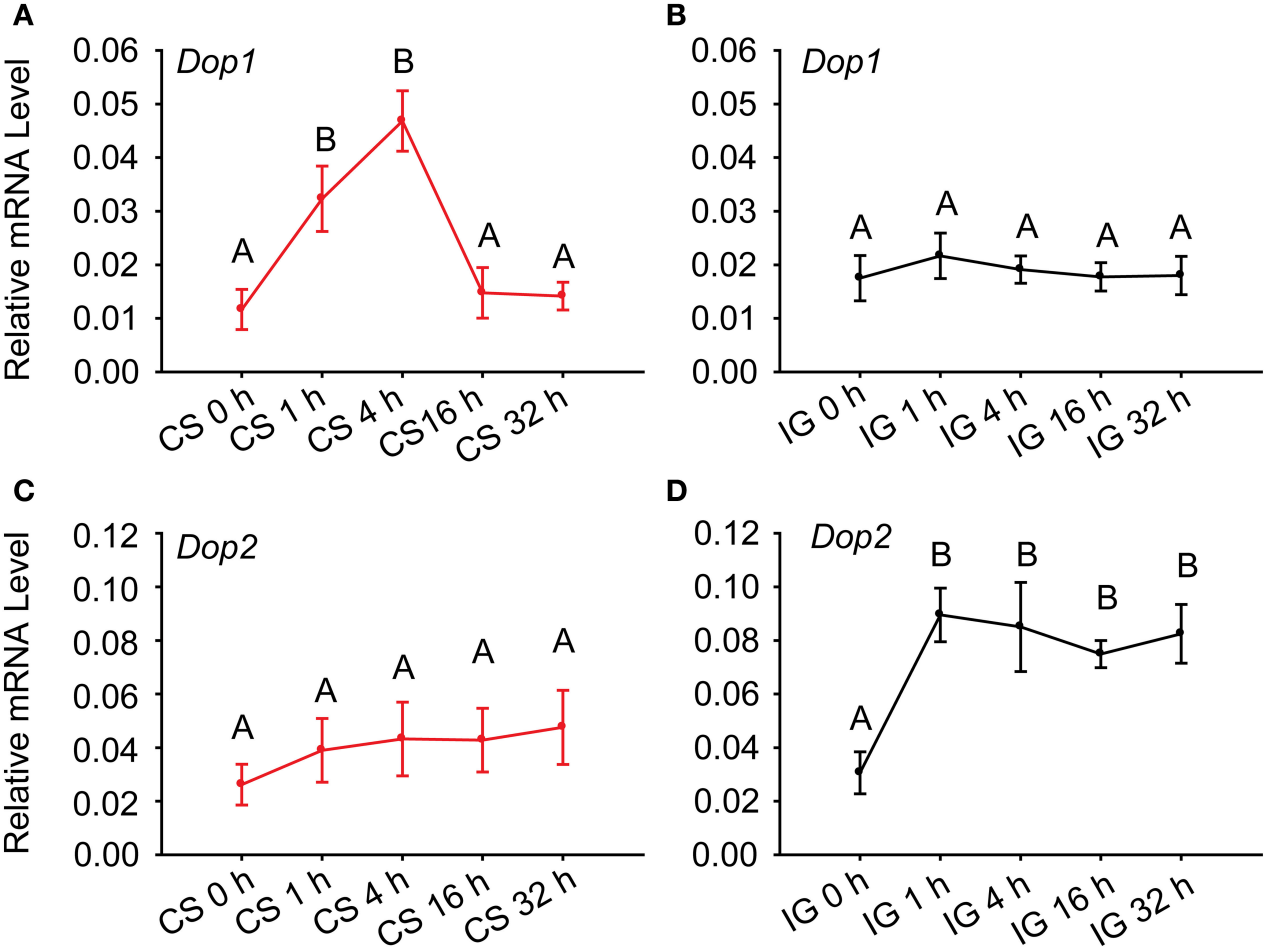
**Expression patterns of *Locusta* Dop1 and Dop2 in the brains of migratory locust during the time course of crowding and isolation. (A)** Level of *Locusta* Dop1 mRNA in the brains of solitary locusts in the time course of crowding. **(B)** Level of *Locusta* Dop1 mRNA in the brains of gregarious locust in the time course of isolation. **(C)** Level of *Locusta* Dop2 mRNA in the brains of solitary locusts in the time course of crowding. **(D)** Level of *Locusta* Dop2 mRNA in the brains of gregarious locust in the time course of isolation. Levels of *Locusta* Dop1 and Dop2 mRNA in crowding and isolation were analyzed with One-Way ANOVA, followed by *post-hoc* Tukey test for multiple comparisons (*n* = 8 for each point). Abbreviations: CS, crowding of solitary locusts; IG, isolation of gregarious locusts.

To further determine the association of dopamine receptors with the behavioral phase change of *Locusta*, we detected their expressions during crowding and isolation. qRT-PCR analysis identified that the expressions of Dop1 mRNA in solitary locusts increased five-fold after 4 h of crowding (One-Way ANOVA, *F* = 10.438, *P* < 0.001) (Figure 3A). However, the mRNA expression level of this receptor did not change during the isolation (One-Way ANOVA, *F* = 0.993, *P* = 0.447) (Figure 3B). By contrast, the expression of Dop2 mRNA remained relatively stable (One-Way ANOVA, *F* = 0.422, *P* = 0.831) during crowding (Figure 3C), significantly increased after 1 h of isolation, and remained relatively stable thereafter (One-Way ANOVA, *F* = 6.910, *P* = 0.001) (Figure 3D). These results suggest that DA-Dop1 signaling is positively associated with gregarization, whereas DA-Dop2 signaling is positively related to solitarization of *Locusta*.

### DA-Dop1 signaling modulates gregarious behavior

**Figure 4:**
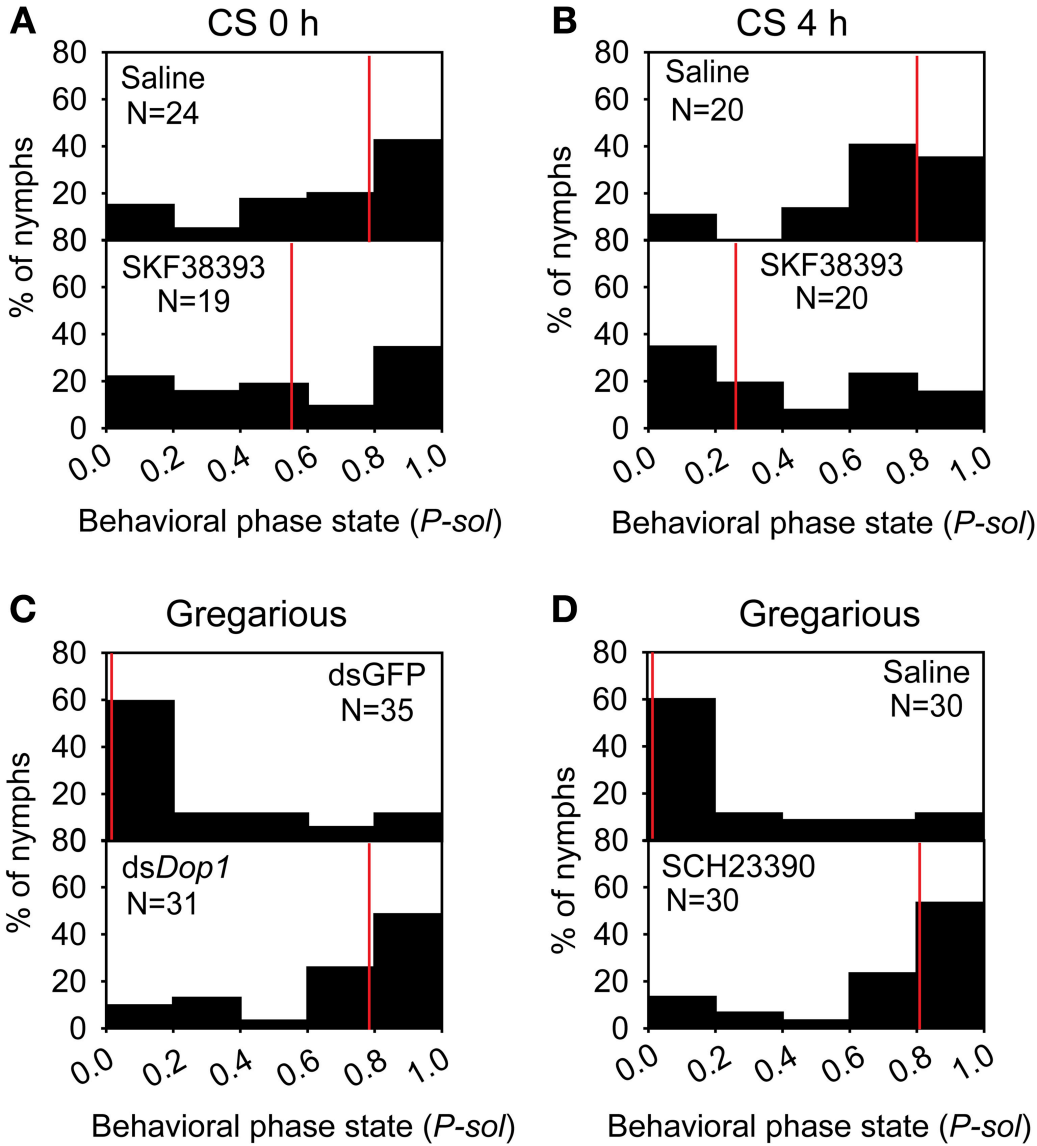
***Locusta* Dop1 promotes the gregariousness of solitary locusts. (A)** The behavioral phase state of solitary locusts after injection of Dop1 agonist, SKF38393. **(B)** The behavioral phase state of crowded solitary locusts after injection of Dop1 agonist, SKF38393. **(C)** The behavioral phase state of gregarious locusts after RNAi knockdown of *Locusta Dop1*. **(D)** The behavioral phase state of gregarious locusts after injection of Dop1 antagonist, SCH23390. The behavioral comparison between controls and the treatments were analyzed by Mann–Whitney *U*-test (*P* < 0.05). Red lines indicate the medians of *P*-*sol* values. *P*-*sol*, probabilistic metric of solitariness. Abbreviations: CS, crowding of solitary locusts.

The up-regulation of DA concentration and *Locusta* Dop1 expression in the brains of solitary locusts after 4 h of crowding leads us to explore the roles of *Locusta* Dop1 in regulating locust gregarization. Thus, we injected SKF38393, the specific agonist of D1-like receptor, into the brains of the migratory locust and detected its effects on behavioral changes after 4 h. The behavioral assay showed that solitary locusts changed their behaviors toward gregarious phase [Mann–Whitney *U* (MWU) test, *U* = 119, *P* = 0.008, SKF38393 vs. saline] (Figure 4A). When coupled with 4 h of crowding, SKF38393 injection induced more pronounced behavioral change toward the gregarious phase (MWU, *U* = 119, *P* = 0.008, SKF38393 with CS 4 h vs. saline with CS 4 h) (Figure 4B). We also examined the behavioral state of gregarious locusts after blocking Dop1 signaling through RNAi knockdown and specific antagonist injection in brain tissue. The injection of *Locusta* Dop1 dsRNA into the brains of solitary locusts resulted in approximately 40% reduction of Dop1 expression level (Student's *t*-test, *t* = 3.068, *P* = 0.008) (Supplementary Figure 3A), and RNAi knockdown of *Dop1* in gregarious locusts induced their behavioral change to solitary phase (MWU, *U* = 231, *P* < 0.001, ds*Dop1* vs. dsGFP) (Figure 4C). In addition, injection of SCH23390, the specific antagonist of Dop1 receptors, into the brains of gregarious locusts, induced their significant behavioral change to the solitary phase (MWU, *U* = 190, *P* < 0.001, SCH23390 vs. saline) (Figure 4D). These results indicate that DA-Dop1 signaling mediates gregarization of *Locusta*.

**Figure 5:**
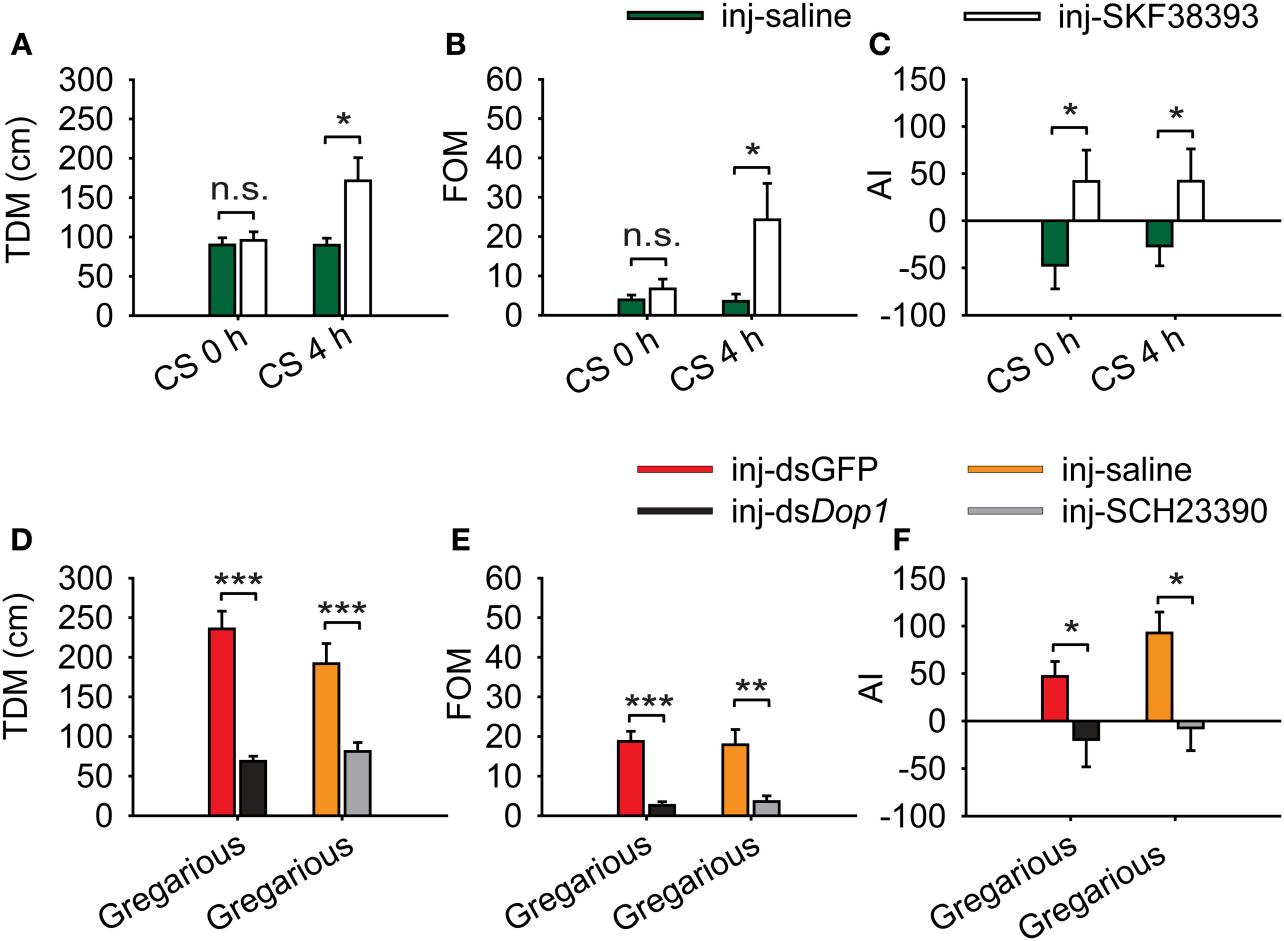
**Change in behavioral markers after activation and inhibition of *Locusta* Dop1. (A)** Change in total distance moved (TDM) of solitary and crowded solitary locusts after injection of SKF38393. **(B)** Change in frequency of movement (FOM) of solitary and crowded solitary locusts after injection of SKF38393. **(C)** Change in attraction index (AI) of solitary and crowded solitary locusts after injection of SKF38393. **(D)** Change in total distance moved (TDM) of gregarious locusts after RNAi knockdown of Dop1 and injection of SCH23390. **(E)** Change in frequency of movement (FOM) of gregarious locusts after RNAi knockdown of Dop1 and injection of SCH23390. **(F)** Change in attraction index (AI) of gregarious locusts after RNAi knockdown of Dop1 and injection of SCH23390. Comparisons of specific behavior markers between controls and the treatments were analyzed by Student's *t*-test **(A,B,D,E)** and Mann–Whitney *U-*test **(C,F)**. *P < 0.05; **P < 0.01; ***P < 0.001; n.s., not significant. Abbreviations: CS, crowding of solitary locusts.

We applied a logistic regression model that encapsulates three behavioral markers to evaluate the change of behavioral phase on the entire level. To explore the behavioral changes after injecting Dop1 agonist, we analyzed the change of the specific behavioral parameters: total distance moved (TDM), frequency of movement (FOM), and attraction index (AI) in phase change. Injection of SKF38393 into the brains of solitary locusts did not increase their TDM and FOM, but induced the shift from avoidance to approaching the stimulus locusts (the gregarious locusts in one of the end chambers in the assay arena) (Student's *t*-test, *t* = 1.795, *P* = 0.078 for TDM; *t* = 1.925, *P* = 0.062 for FOM; MWU, *U* = 265, *P* = 0.039 for AI) (Figures 5A–C). By contrast, when coupled with 4 h of crowding, the injection of SKF38393 increased TDM and FOM of the solitary locusts (Student's *t*-test: *t* = 2.519, *P* = 0.020 for TDM; *t* = 2.494, *P* = 0.022 for FOM) (Figures 5A,B) and induced their attraction toward the stimulus locusts (MWU, *U* = 359, *P* = 0.028) (Figure 5C). Thus, DA-Dop1 signaling in the brains not only mediated their attraction-response to gregarious conspecifics, but also modulated the motility during the crowding of solitary locusts.

RNAi knockdown of *Locusta* Dop1 mRNA in the brains of gregarious locusts significantly reduced their TDM and FOM, as well as induced the shift from approaching to avoiding the stimulus locusts (Student's *t*-test, *t* = 7.205, *P* < 0.001 for TDM; *t* = 5.958, *P* < 0.001 for FOM; MWU, *U* = 244, *P* = 0.015 for AI) (Figures 5D–F). Similarly, injection of SCH23390 in gregarious locusts reduced their TDM and TOM, as well as induced the avoidance to the stimulus locusts (Student's *t*-test, *t* = 3.986, *P* < 0.001 for TDM; *t* = 3.442,*P* = 0.001 for FOM; MWU, *U* = 348, *P* = 0.018 for AI) (Figures 5D–F).

### DA-Dop2 signaling mediates solitary behavior

**Figure 6:**
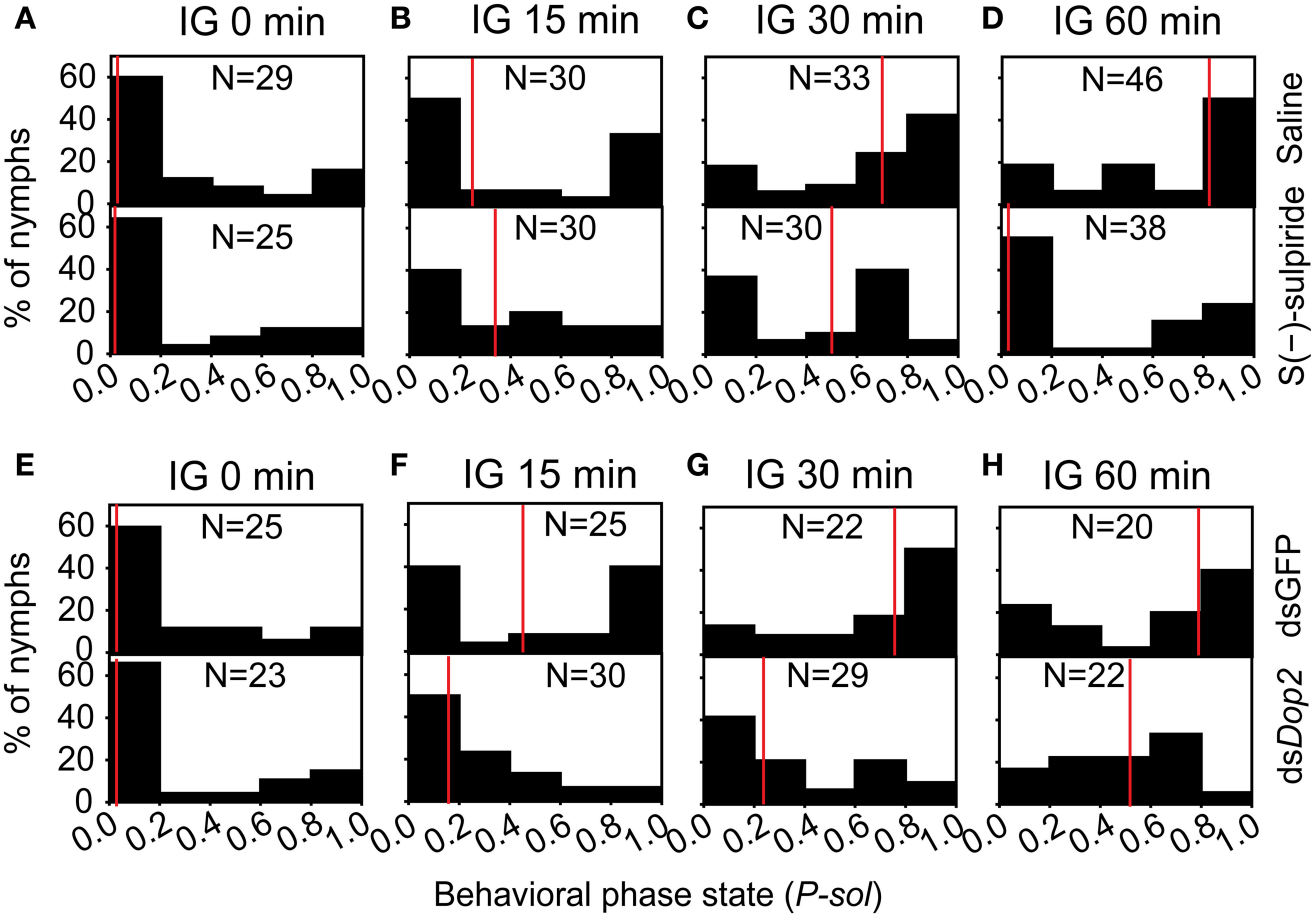
**Behavioral change in isolated gregarious locusts with deficiency of *Locusta* Dop2 signaling. (A)** Behavioral state of gregarious locusts after injection of Dop2 antagonist S(-)-sulpiride. **(B–D)** Behavioral state of gregarious locusts with 15 min **(B)**, 30 min **(C)**, and 60 min **(D)** of isolation after injection of S(-)-sulpiride. **(E)** Behavioral state of gregarious locusts after RNAi knockdown of *Locusta* Dop2. **(F–H)** Behavioral state of gregarious locusts with 15 min **(F)**, 30 min **(G)**, and 60 min **(H)** of isolation after RNAi knockdown of *Locusta* Dop2. The behavioral comparison between controls and the treatments were analyzed by Mann–Whitney *U*-test (*P* < 0.05). Red lines indicate the medians of *P*-*sol* values. Abbreviations: IG, isolation of gregarious locusts; *P*-*sol*, probabilistic metric of solitariness.

During the isolation of the migratory locust, the level of DA decreased, whereas the expression of *Locusta* Dop2 mRNA significantly increased. These results suggest that *Locusta* Dop2 mediated the solitarization of *Locusta*. To explore this, we injected S(-)-sulpiride, the antagonist of Dop2, in the brains of gregarious locusts and detected its effects on their behavioral state. The injection of S(-)-sulpiride into the brains of gregarious locusts (IG 0 min) did not change their behavioral phase (MWU, *U* = 351, *P* = 0.834) (Figure 6A). After 15 min of isolation, the saline-injected gregarious controls showed behavioral change toward the solitary phase (MWU, *U* = 249, *P* = 0.005), but significant difference was not observed between saline-injected gregarious controls and S(-)-sulpiride-injected gregarious locusts (MWU, *U* = 367, *P* = 0.222) (Figure 6B). After 30 or 60 min of isolation, the injection of S(-)-sulpiride significantly inhibited the behavioral change of the isolated gregarious locusts toward the solitary phase (MWU, *U* = 307, *P* = 0.010 for 30 min; *U* = 534, *P* = 0.002 for 60 min), despite that the gregarious controls injected with saline exhibited the solitary-like behavior compared with gregarious locusts (MWU, *U* = 162, *P* < 0.001 for 30 min; *U* = 245, *P* < 0.001 for 60 min) (Figures 6C,D).

To avoid the off-target effects of Dop2 antagonist, we cloned a fragment of *Dop2* and designed the dsRNA to specifically knockdown the expression of *Locusta* Dop2 mRNA for functional validation. The injection of *Locusta* Dop2 dsRNA in the brains of solitary locusts resulted in approximately 50% reduction of *Locusta* Dop2 mRNA level (Student's *t*-test, *t* = 6.142, *P* < 0.001) (Supplementary Figure 3B), whereas no effects on the gregarious behaviors was observed (MWU, *U* = 256, *P* = 0.514) (Figure 6E). As above, after 15, 30, or 60 min of isolation, the gregarious locusts injected with dsGFP showed behavioral shift toward solitary phase (MWU,*U* = 174, *P* = 0.007 for 15 min; *U* = 93, *P* < 0.001 for 30 min; *U* = 93, *P* < 0.001 for 60 min), whereas the deficiency of *Locusta* Dop2 inhibited the behavioral change of gregarious locusts toward solitary phase, as compared with the corresponding dsGFP-injected controls (MWU, *U* = 201, *P* = 0.003 for 15 min; *U* = 137, *P* = 0.001 for 30 min; *U* = 100, *P* = 0.019 for 60 min) (Figures 6F–H).

On the other hand, we activated *Locusta* Dop2 in solitary locusts through injection of Dop2 specific agonist, and detected their behavioral state after 60 min. However, the reported agonists in insects will activate both Dop1 and Dop2 (Feng et al., 1996; Blenau et al., 1998; Weisel-Eichler et al., 1999; Mustard et al., 2003), or are almost ineffective (Feng et al., 1996). Thus, we selected another vertebrate D2-receptor selective agonist, R(-)-TNPA, to examine its effect on behavioral phase state of solitary locust. The results showed that injection of R(-)-TNPA into the brain of solitary locusts did not affect their behavioral phase state (MWU, *U* = 306, *P* = 0.327) (Supplementary Figure 4).

**Figure 7:**
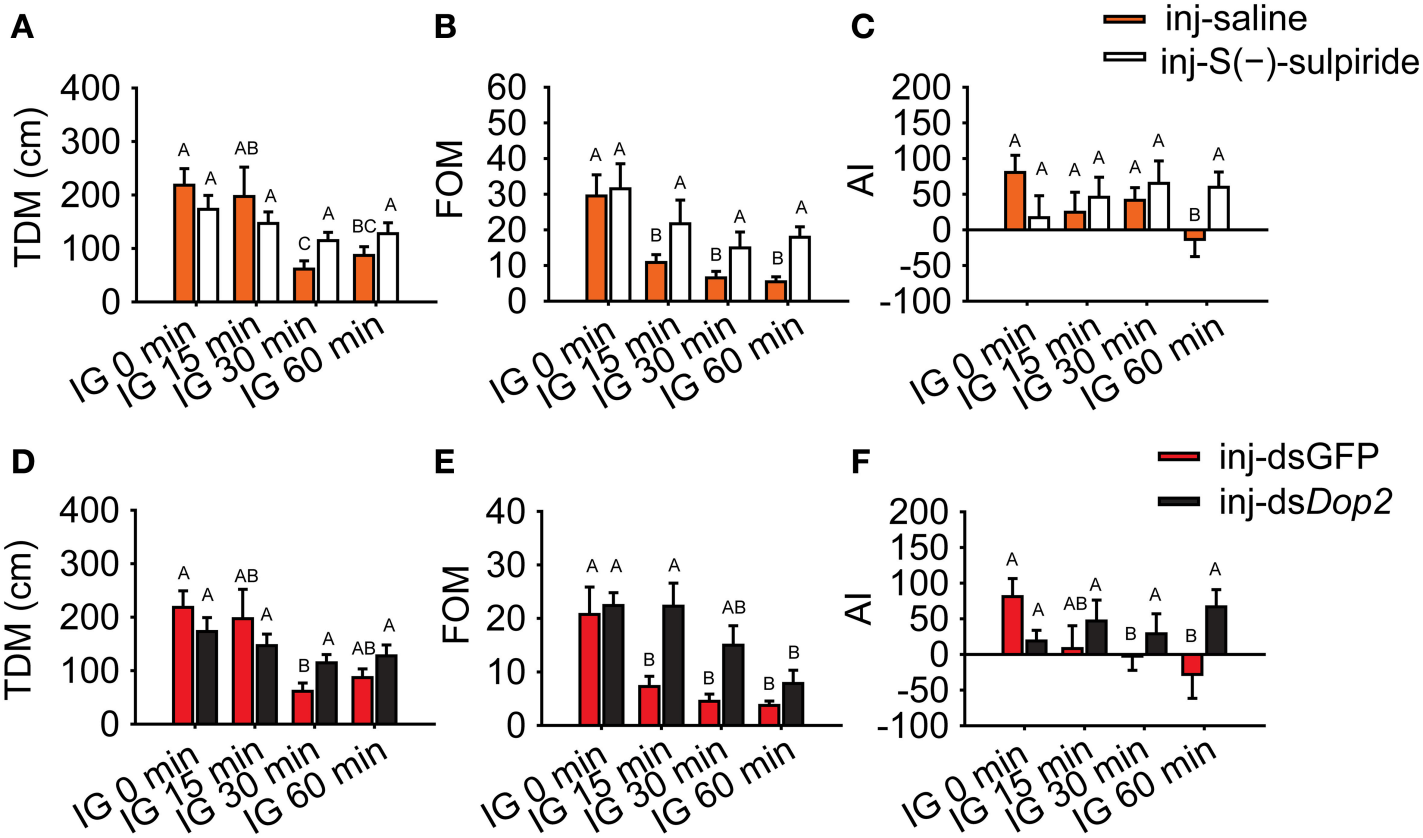
**Change in behavioral markers in isolated gregarious locusts with Dop2 blockade. (A)** Changes in total distance moved (TDM) in isolated gregarious locusts after injection of Dop2 antagonist, S(-)-sulpiride. **(B)** Changes in frequency of movement (FOM) in isolated gregarious locusts after injection of S(-)-sulpiride. **(C)** Changes in attraction index (AI) in isolated gregarious locusts after injection of S(-)-sulpiride. **(D)** Changes in total distance moved (TDM) in isolated gregarious locusts after RNAi knockdown of *Locusta* Dop2. **(E)** Changes in frequency of movement (FOM) in isolated gregarious locusts after RNAi knockdown of *Locusta* Dop2. **(F)** Changes in attraction index (AI) in isolated gregarious locust after RNAi knockdown. The comparison between controls and the treatments **(A, B, D, E)** was analyzed with One-Way ANOVA followed by *post-hoc* Tukey test for multiple comparisons. The behavioral comparison between controls and treatments **(C, F)** was analyzed by Mann–Whitney *U*-test. Abbreviations: IG, isolation of gregarious locusts.

Moreover, we evaluated the logistic regression model and specifically analyzed the encapsulated behavioral parameters. During isolation, TDM and FOM in gregarious locusts significantly decreased (One-Way ANOVA, *F* = 6.331, *P* < 0.001 for TDM; *F* = 13.381, *P* < 0.001 for FOM), whereas changes were not observed after Dop2 antagonist was injected (One-Way ANOVA, *F* = 1.315, *P* = 0.273 for TDM; *F* = 1.736, *P* = 0.273 for FOM) (Figures 7A,B). The AI analysis showed that gregarious controls significantly avoided the stimulus locusts after 60 min of isolation (MWU, *U* = 415, *P* = 0.006, saline-injected gregarious locusts with IG 60 min vs. saline-injected gregarious with IG 0 min), whereas the antagonist-injected groups tended to approach the stimulus locusts (MWU, *U* = 346, *P* = 0.405, antagonist-injected gregarious with IG 60 min vs. antagonist-injected gregarious with IG 0 min) (Figure 7C).

Similarly, TDM in RNAi-knockdown groups remained stable during the time course of isolation (One-Way ANOVA, *F* = 2.246, *P* = 0.086) compared with the dsGFP-injected groups, which displayed a significant decrease in TDM during isolation (One-Way ANOVA, *F* = 5.777, *P* = 0.001) (Figure 7D). The isolated gregarious controls showed significant reduction in FOM after 15 min of isolation (One-Way ANOVA, *F* = 8.095, *P* < 0.001). However, the deficiency of Dop2 signaling caused by RNAi-knockdown inhibited the decrease in FOM, and the treated gregarious locusts showed significant decrease in FOM after 60 min of isolation (One-Way ANOVA, *F* = 4.046, *P* = 0.009) (Figure 7E). After 60 min of isolation, dsGFP-injected gregarious controls significantly avoided the stimulus locusts (MWU, *U* = 225, *P* = 0.011, dsGFP-injected gregarious with IG 60 min vs. dsGFP-injected gregarious with IG 0 min), whereas the ds*Dop2*-injected groups tended to approach the stimulus locusts (MWU, *U* = 414, *P* = 0.900, ds*Dop2*-injected gregarious with IG 60 min vs. ds*Dop2*-injected gregarious with IG 0 min) (Figure 7F). In solitary locusts, injection of R(-)-TNPA did not affect their TDM, FOM and their response to the stimulus locusts (Student's *t*-test, *t* = 0.430, *P* = 0.354 for TDM; *t* = 0.277, *P* = 0.783 for FOM; MWU, *U* = 315, *P* = 0.384 for AI) (Supplementary Figure 5). These results suggest that *Locusta* Dop2 induced the solitarization of *Locusta* by decreasing motility and inhibiting conspecific attraction.

## Discussion

In this study, we found two dopamine receptors playing different roles in phase change of *Locusta*. The increased DA level and up-regulation of *Locusta Dop1* expression in crowding suggested their correlations with the gregarization of *Locusta*, and we verified that dopamine-Dop1 signaling mediated behavioral gregariousness. Moreover, the increased expression of *Locusta Dop2* in isolation suggested its correlations with solitarization, and we confirmed that dopamine-Dop2 signaling mediated the behavioral solitariness in *Locusta*. This study clarified the different functions of dopamine receptors underlying behavioral phase change in *Locusta*.

*Locusta* Dop1 mediated the gregariousness of *Locusta* by inducing the attraction-response and increasing motility, and *Locusta* Dop2 regulated the solitariness of *Locusta* by inducing the repulsion-response and decreasing the motility, suggesting that DA-Dop1 and DA-Dop2 signaling mediate sensory interaction between conspecifics during aggregation. A number of studies have suggested that dopamine mediates the sensory perception of vertebrates and invertebrates, such as olfaction (Kim et al., 2007; Serguera et al., 2008), taste (Cannon et al., 2005), vision (Jackson et al., 2012), and auditory (Li et al., 2013). Specifically, dopamine is reported to mediate social interactions in insects. A study on crickets has found that DA involves the shift from avoiding conspecific males to agonistic dispute with conspecifics during the recovery of aggression after social defeat (Rillich and Stevenson, 2014). In the antenna of worker bees (*Apis mellifera* L.), AmDOP3 receptors regulate their attraction-response to the queen mandibular pheromone (QMP) (Vergoz et al., 2009). In ants (*Harpegnathos saltator*), the fluctuation of DA levels in brains and ovaries are respectively associated with ritualized combat and the formation of a reproductive hierarchy (Penick et al., 2014). Therefore, dopamine may integrate the outer stimuli for the decision to join the group or conspecific withdrawal through *Locusta* Dop1 and Dop2, respectively.

The level of DA, the expression, and the functional properties of *Locusta* Dop1 and Dop2 may also contribute to their divergent functions. *Locusta* Dop1 and Dop2, which belong to D1-like receptors and INDRs, both activate the cAMP-PKA signaling (Beggs et al., 2005; Mustard et al., 2005). In *Schistocerca*, cAMP-dependent protein kinases (PKA) is critical in the acquisition of the gregarious behavior (Ott et al., 2012). Besides linking with 5-HT signaling in mediating the crowding of *Schistocerca* (Ott et al., 2012), PKA is also involved in DA signaling (Romanelli et al., 2010; Mustard et al., 2012). Thus, the higher level of cAMP-PKA may be related to the gregarization in the two locust species. In *Locusta*, cAMP-PKA signaling may be greatly activated by the increasing of DA level and Dop1 mRNA expression in crowding, and the up-regulation of cAMP-PKA signaling promotes the locust gregarization. During isolation, the tendency of dopamine level in isolation is contray to the expression of Dop2 mRNA. The decrease in dopamine concentration in the brain will reduce the cAMP-PKA level, and the increasing of Dop2 expression may regulate other downstream signaling and finally result in quick solitarization in *Locusta*. Thus, the tendency of DA concentration and the expression and functional properties of *Locusta* Dop1 and Dop2 may result in the divergence of these two receptors underlying locust phase change.

In *Schistocerca*, injection of DA into the thoracic cavities of gregarious locusts induces solitarization and avoidance behavior (Alessi et al., 2014). This result is similar to that obtained in our study where DA-Dop2 signaling in brains mediated solitarization and conspecific avoidance-response in the migratory locust. However, the study in *Schistocerca* did not point out that dopamine mediates solitarization through Dop1 or Dop2, or other receptor subtypes simultaneously. Meanwhile, the non-selective antagonist fluphenazine (Degen et al., 2000) in *Schistocerca* has been employed for administrations to confirm the function of dopamine (Alessi et al., 2014). We cannot definitely clarify whether the mediation of solitarization and avoidance behavior by dopamine in the two locust species are through identical or different mechanisms. Moreover, the function of dopamine mediating solitarization of *Schistocerca* is contrary to the function of *Locusta* Dop1 in the gregarization of *Locusta*. The applications of 5-HT also lead to distinct effects on phase change of *Schistocerca* from different laboratories and *Locusta* (Anstey et al., 2009; Tanaka and Nishide, 2012; Guo et al., 2013). These results would partially be attributed to their species-specific traits because *Locusta* and *Schistocerca* belong to different Acrididae subfamilies, Cyrtacanthacridinae and Oedipodinae, respectively.

The function of dopamine in regulating locust phase change may be closely correlate with the target tissues where dopamine is located and where its function is apparent. In *Locusta*, the fluctuations and functions of DA and its receptors were analyzed and verified in the brain, whereas in *Schistocerca*, the investigation of dopamine function in phase change mainly focused on the thoracic ganglia. In *Schistocerca*, the application of DA induced a significant reduction in the amplitude of the CS-FETi EPSP in the metathoracic ganglia, and the systematic injection of dopamine in gregarious locust induced more behavioral avoidance (Alessi et al., 2014). However, the systematic injection of dopamine into the thoracic cavity may merge its effects in the brain and thoracic ganglia. The brain of an insect can integrate multisensory inputs and direct patterns of activity ascended by “lower” neural centers, such as thoracic ganglia and abdominal ganglia (Schaefer and Ritzmann, 2001; Zill, 2010), and many complex and important behaviors of insects are integrated and controlled by the brain (Wessnitzer and Webb, 2006). Many innate behaviors, such as locomotion, feeding, and mating, are controlled by body ganglia, but not the brain (Wessnitzer and Webb, 2006). Thus, whether thoracic ganglia can regulate attraction and repulsion behavior through the sensory system in the brain during locust phase change should be fully explored in the future. The present study in *Locusta* suggests that the functions of dopamine in gregarization and solitarization are closely related to the receptor subtypes to which dopamine binds. By contrast, in *Schistocerca*, the expression patterns and functions of different receptor subtypes were unclear; the injection of dopamine and non-selective antagonist in thoracic cavity may merge the functions of multi receptor subtypes in behavioral plasticity.

The functions of dopamine-Dop1 and Dop2 signaling in phase change of *Locusta* were confirmed via pharmacological intervention and RNAi-knockdown. Pharmacological intervention by injecting agonists and antagonists of dopamine receptors may introduce off-target effects and therefore non-specifically activate or inhibit other biogenic amine receptors. Meanwhile, RNA interference, through exogenous introduction of dsRNA with sequences complementary to the targeted genes, provides specific manipulation of gene expression and is widely used to investigate gene functions (Fire et al., 1998; Bosher and Labouesse, 2000; Milhavet et al., 2003). The coupled application of these two methods in the present study verified the different functions of *Locusta* Dop1 and Dop2 during phase change of *Locusta*. By contrast, in *Schistocerca*, pharmacological injection was applied to confirm the function of dopamine in solitarization (Alessi et al., 2014) and this study lacks necessary gene function study, such as RNAi knockdown, to avoid the off-target and non-specificity effects of dopamine antagonists on receptors in phase change of *Schistocerca*. Therefore, genetic information and functional verification of dopamine signaling involved in modulating phase change from *Schistocerca* are very essential, because it will be beneficial for the clarification of the divergence and convergence of the functions of dopamine in phase change of the two locust species.

In conclusion, different neurochemicals and molecular mechanisms are likely to perform on the same behavioral paradigm of transition in different species. With the development of next generation sequencing, dissection of genome sequences in closely related locust species and deep exploration of molecular mechanism of phase change will be helpful in understanding the divergence and convergence of mechanisms underlying the classical phenotypic plasticity in different locust species.

### Conflict of interest statement

The authors declare that the research was conducted in the absence of any commercial or financial relationships that could be construed as a potential conflict of interest.
