# Supplementary material for: Two dopamine receptors play different roles in phase change of the migratory locust
Source: Front Behav Neurosci. 2015 Mar 31;9:80. doi: 10.3389/fnbeh.2015.00080 (PMC4379914; doi:10.3389/fnbeh.2015.00080)
Supplement: Supplementary file 1 [file DataSheet1.DOCX]

**Supplementary material**

**Two dopamine receptors play different roles in phase change of the migratory locust**

Xiaojiao Guo^†^, Zongyuan Ma^†^, Le Kang*

^1^Beijing Institutes of Life Sciences, Chinese Academy of Sciences, Beijing, China

^2^State Key Laboratory of Integrated Management of Pest Insects and Rodents, Institute of Zoology, Chinese Academy of Sciences, Beijing, China

*Correspondence: Le Kang, Ph.D., Professor of Entomology, State Key Laboratory of Integrated Management of Pest Insects and Rodents, Institute of Zoology, Chinese Academy of Sciences, Beichen West Road, Chaoyang District, Beijing 100101, China E-mail: lkang@ioz.ac.cn

**Supplementary Figures**


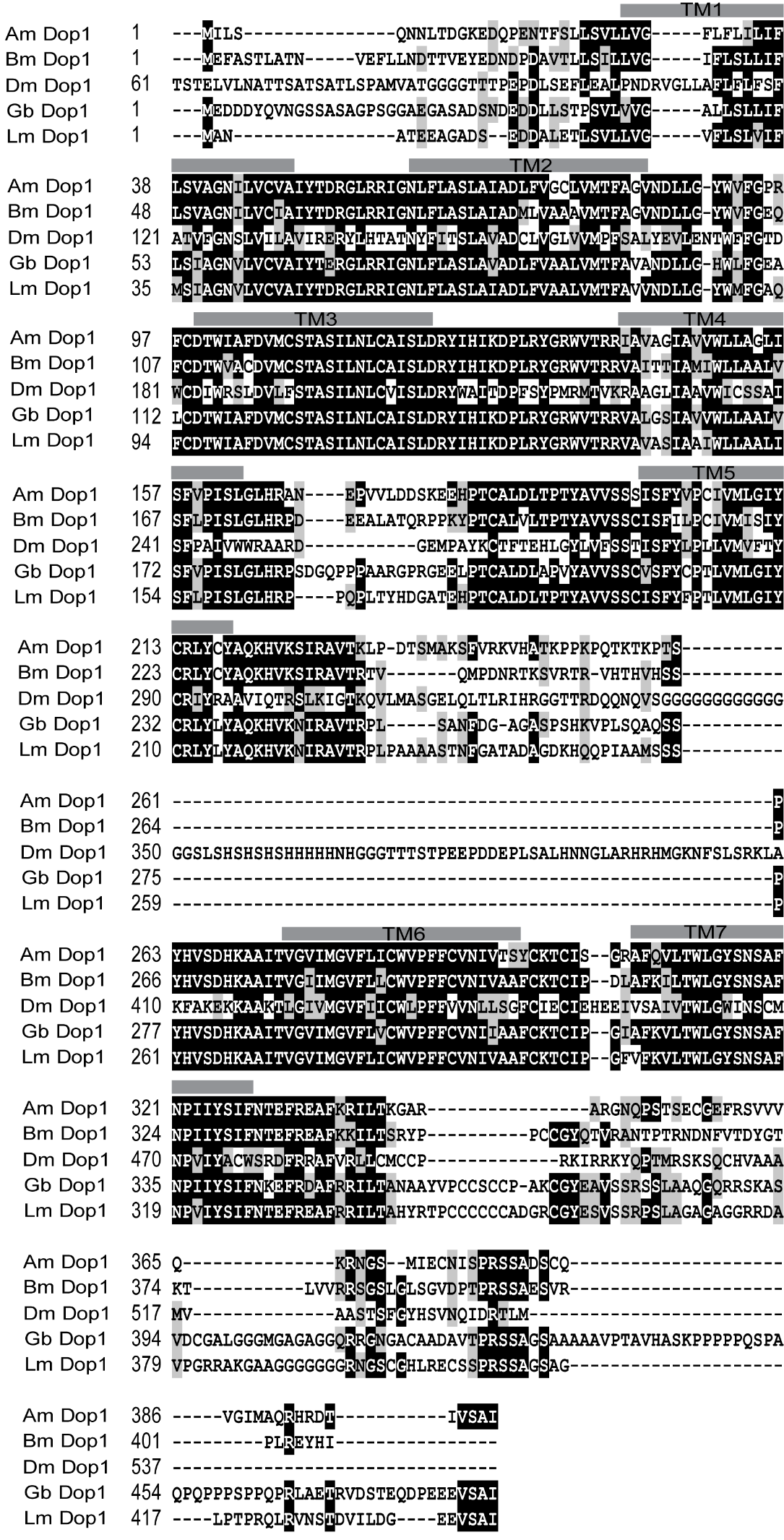


**Supplementary Figure 1.** **Amino acid sequence alignment of dopamine receptor 1 (Dop1) in *Locusta migratoria* and the ones in other insects.** (A) Alignment of the amino acid sequences of LmDop1 (Genbank accession number: KP780182) with orthologous receptors from *Apis mellifera* (AmDop1, CAA73841), Bombyx mori (BmDop1, NP_001108459), *Drosophila melanogaster* (DmDop1, AAB08000), and *Gryllus bimaculatus* (GbDop1*,* BAM15634). Identical residues of the aligned sequences are shown as white letters against black ones, whereas conservatively substituted residues are shaded. Putative transmembrane regions are indicated by gray bars. Dashes indicate gaps that were introduced to maximize homology.


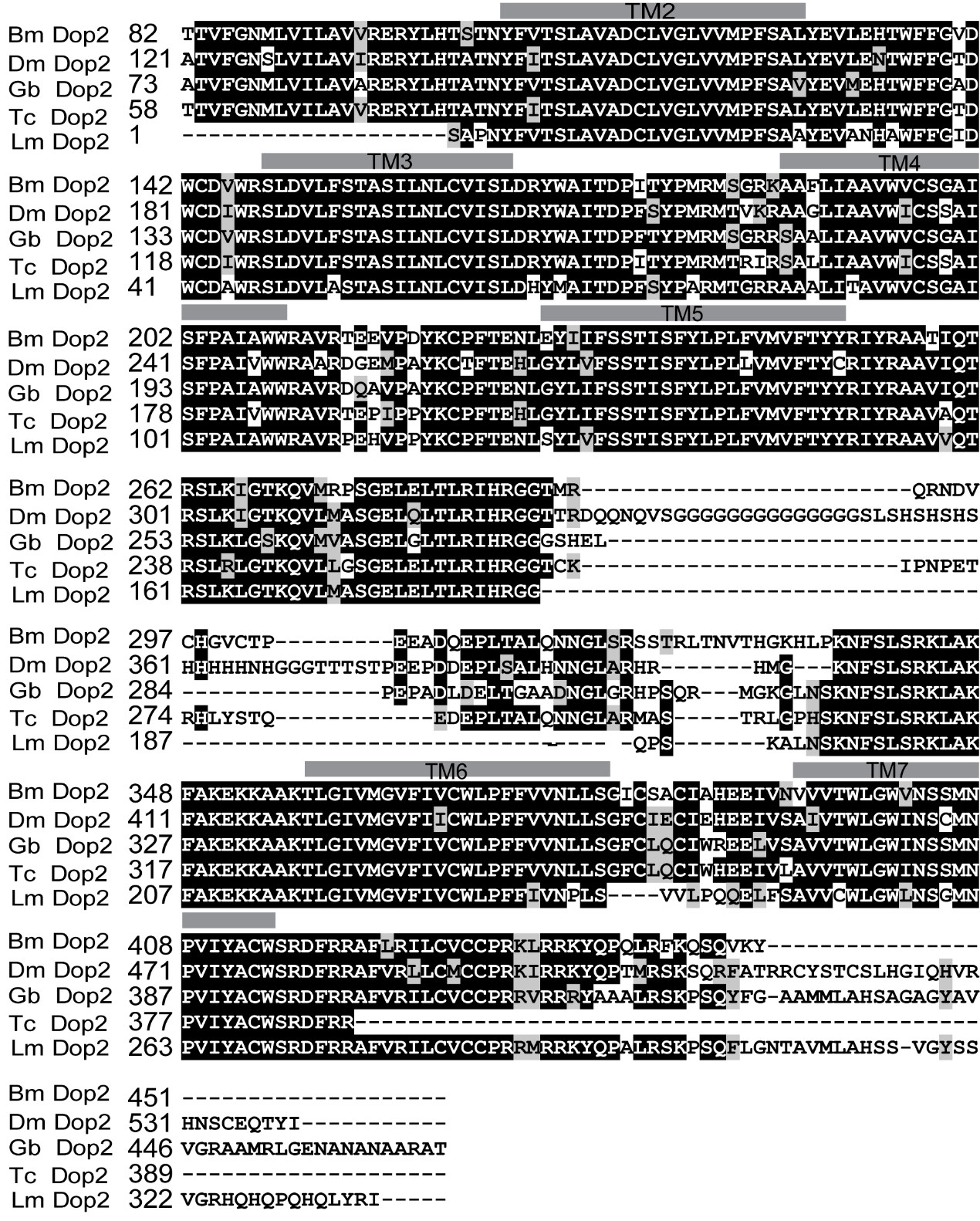


**Supplementary Figure 2. Amino acid sequence alignment of dopamine receptor 2 (Dop2) in *Locusta migratoria* and the ones in other insects.** (A) Alignment of the amino acid sequences of LmDop2 (Genbank accession number: KP780183) with orthologous receptors from *Bombyx mori* (BmDop2, NP_001108338), *Drosophila melanogaster* (DmDop2, NP_524548), *Gryllus bimaculatus* (GbDop2*,* BAM15635), and *Tribolium castaneum* (TcDop2, XP_972779). Identical residues of the aligned sequences are shown as white letters against black ones, whereas conservatively substituted residues are shaded. Putative transmembrane regions are indicated by gray bars. Dashes indicate gaps that were introduced to maximize homology.


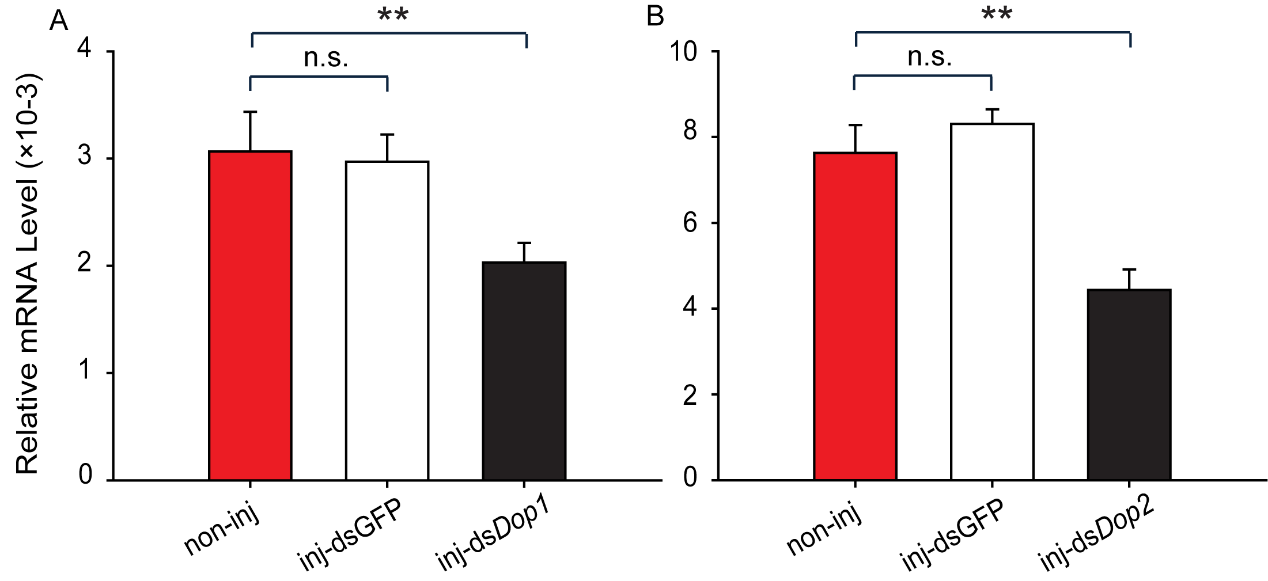


**Supplementary Figure 3. Effects of RNAi knockdown on the relative mRNA level of Dop1 and Dop2 in the brain of *Locusta*.** The data represent mean values and error bars represent SEM. The treatment (inj-dsGFP, inj-ds*Dop1* or inj-ds*Dop2*) compared with the corresponding non-injected controls (Student’s *t*-test). **, *P* < 0.01; n.s., not significant.


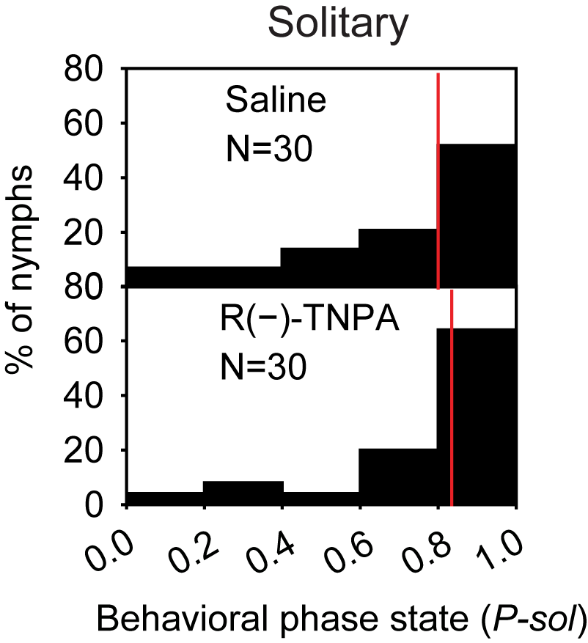


**Supplementary Figure 4. Behavioral change in solitary locusts with activation of Dop2.** The behavioral phase state of solitary locusts after injection of Dop2 agonist, R(−)-TNPA. The behavioral comparison between R(−)-TNPA-injected groups and saline-injected groups were analyzed by Mann–Whitney U test. Red lines indicate the medians of *P-sol* values. *P-sol*, probabilistic metric of solitariness.


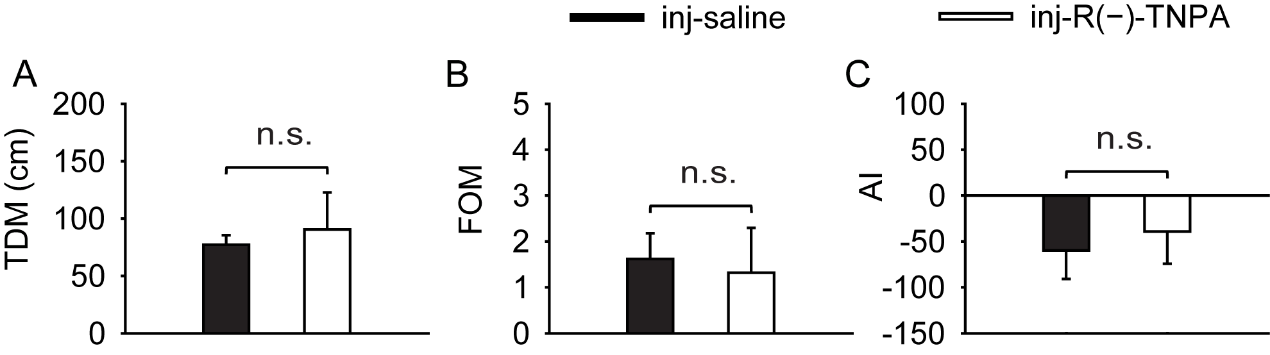


**Supplementary Figure 5. Change in behavioral markers in solitary locusts with Dop2 activation.** (A) Change in total distance moved (TDM) in solitary locusts after injection of Dop2 agonist, R(−)-TNPA. (B) Change in frequency of movement (FOM) in solitary locusts after injection of Dop2 agonist, R(−)-TNPA. (C) Change in attraction index (AI) in solitary locusts after injection of Dop2 agonist, R(−)-TNPA. Comparisons of specific behavior markers between the groups of inj-R(−)-TNPA and inj-saline were analyzed by Student’s *t*-test (A and B) and Mann–Whitney U test (C). n.s., not significant.
